# Kelvin Probe Force Microscopy Study of a Pt/TiO_2_ Catalyst Model Placed in an Atmospheric Pressure of N_2_ Environment

**DOI:** 10.1002/asia.201101001

**Published:** 2012-04-18

**Authors:** Ryohei Kokawa, Masahiro Ohta, Akira Sasahara, Hiroshi Onishi

**Affiliations:** a Advanced Measurement and Analysis Project, Japan Science and Technology AngencyHoncho, Kawaguchi, Saitama, 332-0012 Japan; b Shimadzu CorporationNakagyo, Kyoto, 604-8511 Japan E-mail: kokawa@shimadzu.co.jp; c Department of Chemistry, Kobe UniversityRokko-dai, Nada, Kobe, Hyogo, 657-8501 Japan E-mail: oni@kobe-u.ac.jp; d School of Material Science, Japan Advanced Institute of Science and TechnologyAsahidai, Nomi, Ishikawa, 923-1292 Japan

**Keywords:** electron transfer, heterogeneous catalysis, nanotechnology, scanning probe microscopy, supported catalysts

## Abstract

A catalyst model comprising platinum nanoparticles deposited on a TiO_2_(110) wafer was prepared in a vacuum, transferred in air, and characterized with a Kelvin probe force microscope placed in a N_2_ environment. The topography and local work function of individual nanoparticles were observed with single-nanometer resolution in the N_2_ environment of one atmosphere pressure. Some nanoparticle presented positive shifts of work function relative to that of the TiO_2_ surface, while the others showed negative shifts. This finding suggests heterogeneous properties of the nanoparticles exposed to air and then N_2_. The ability of the advanced microscope was demonstrated in observing the work function of metal nanoparticles on a metal oxide support even in the presence of vapor environments.

## Introduction

Our current civilization is supported by a number of chemical processes for artificial materials production. Heterogeneous catalysts are solid-state devices that assist the chemical reactions of demand. Most catalysts contain nanometer-sized transition-metal particles interfaced with metal oxide supports. The charge transfer from metal to metal oxide is thought to affect reactions catalyzed on the nanoparticles. Fine tuning of catalyst activity and selectivity has been achieved by using particle–support charge transfer. X-ray photoelectron spectroscopy provides an efficient method to evaluate the extent of charge transfer, based on the chemical shift of core-level photoemission. While the observed chemical shifts are averaged over the catalyst, the metal particles are not always homogeneous. Particles can be of different sizes or interfaced with different sites of the support, for example, terraces, steps, or kinks. The composition of adsorbed species should also be heterogeneous nanoparticle-by-nanoparticle. When the transferred charge is quantified on each nanoparticle, much progress can be made in the methods of catalyst characterization. Application of Kelvin probe force microscopy (KPFM) has been proposed for this purpose.[1], [2]

**Figure 1:**
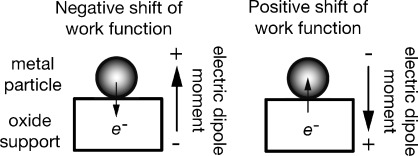
Work function shifts induced by electron transfer at nanoparticle–support interfaces.

When a transition-metal nanoparticle donates electrons to the support, an electric dipole moment appears at the interface. The moment is directed from the support to the nanoparticle. The work function of the support is reduced by the outward-directed dipole moment (Figure 1), since the dipole moment acts as a miniaturized electric double layer. Hence the work function presents a local, negative shift over the electron-donating particle. In contrast, a positive shift of local work function is expected with an electron-accumulating particle. The particle-induced local shifts of work function can be observed by KPFM.

As described below, a number of researchers including the authors have demonstrated single-nanometer or atomic resolution with KPFM operated in a vacuum, where microscopes present their best performance. However, catalyst characterization should be done in vapor atmospheres where catalysts work. High-resolution imaging in practical pressures remains a challenge to applications of KPFM. In the current study, we modified a commercial AFM instrument to enhance the signal-to-noise ratio and applied the modified microscope to KPFM observation in a N_2_ environment of one atmospheric pressure. The object under consideration was Pt nanoparticles deposited on rutile TiO_2_(110) surface. Surfaces of single-crystalline rutile[3]–[10] and anatase[11] TiO_2_ with deposited platinum particles have frequently been examined as catalyst models using scanning probe microscopes.

### Kelvin Probe Force Microscopy (KPFM)

Kelvin probe force microscopy[12], [13] is based on frequency-modulation atomic force microscopy (FM-AFM)[14] and simultaneously provides the topography and local work function of a solid object. In FM-AFM, the resonance oscillation of a cantilever is mechanically excited. When conservative force is applied to the tip, the resonance frequency shifts accordingly. The topography of the solid object is traced with regulation of the tip–surface distance by keeping the frequency shift constant. In KPFM, the oscillating tip is used as the miniaturized reference electrode of a Kelvin probe. The tip and surface form a capacitor, and the contact potential difference (CPD) between the two electrodes causes an electrostatic tip–surface force. The strength of the electrostatic force is oscillated by applying an oscillating sample bias voltage (*V*_s_) relative to the tip. When a direct-current (DC) voltage is further added to the oscillated *V*_s_ and compensates for CPD, the oscillated component of tip–surface force disappears. The microscope is operated to find the compensating DC voltage at different places over the object. The obtained map of compensating voltage represents the lateral distribution of CPD and thus the distribution of local work function.

In the low-noise extreme, the lateral resolution of the work function distribution is limited by the radius of the tip apex and by the tip–surface distance.[15]–[17] Single-nanometer or atomic resolution was achieved on semiconductors, Au/Si(111),[18] Si(111),[19] Sb/Si(111),[20] Ge/Si(105),[21] Sb/Si(111),[22] oxidized Si(111),[23] P dopant buried in Si,[24] TiO_2_(110),[25], [26] and alkali halides(001).[27] Successful applications to catalyst-related materials have further been done on WO_3_/TiO_2_,[28] Au/NaCl,[29], [30] Pd/MgO(001),[31] MgO/Au,[32] and Pd/graphite.[2] The authors conducted a series of KPFM studies with TiO_2_(110) modified by Na adatoms,[33] Cl adatoms,[34] Pt adatoms,[8] Pt nanoparticles,[9], [10] and photosensitizer dyes.[35]

KPFM based on FM-AFM requires a triple feedback loop with respect to the cantilever oscillation amplitude, frequency shift, and contact potential difference. The triple feedback loop is more complex and more sensitive to external noise than the double loop of an ordinary FM-AFM instrument. The signal-to-noise ratio is seriously reduced when the cantilever is oscillated in vapor. The vapor-induced viscous resistance negatively affects the quality factor of cantilever oscillation. As a result, the lateral resolution of the work function has been limited to 30 nm in vapor environments of one atmospheric pressure, whereas an atomic or molecular resolution is available in the topography provided by the ordinary FM-AFM in the presence of vapor environments of this pressure.[36]-[38] In the current study, we modified a microscope to enhance the signal-to-noise ratio and demonstrated a single-nanometer resolution of work function distribution under a vapor environment of one atmospheric pressure.

## Results and Discussion

**Figure 2:**
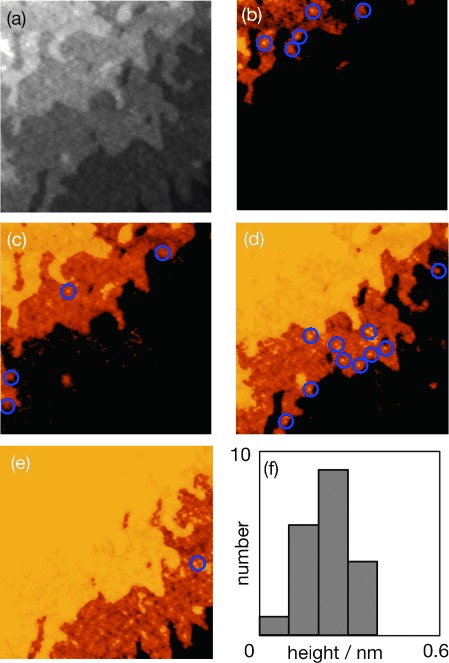
The topography of a catalyst model comprising TiO_2_(110) with deposited Pt observed in a N_2_ atmosphere. A sputter-annealed TiO_2_ wafer was exposed to the Pt source for 20 min. Image size: 300 nm square. Frequency shift set point: −30 Hz. Peak-to-peak amplitude: 5.7 nm. Quality factor of the cantilever oscillation: 400. The raw topography (a) is presented in a simple gray scale. The topography is duplicated and shown in (b) with an adjusted contrast to identify nanoparticles deposited on the top terrace. Platinum nanoparticles are marked with circles in the contrast-adjusted topography. Nanoparticles on the underlying terraces are identified and marked as shown in (c), (d) and (e). The distribution of the particle height is shown in (f).

Figure 2 shows the topography of a catalyst model comprising TiO_2_(110) with deposited Pt. The topography was observed with the ordinary FM-AFM in a N_2_ environment of one atmospheric pressure. The negative set point of frequency shift regulation, −30 Hz, suggested an attractive tip–surface force and thus noncontact scans by the oscillating tip. The quantitative relationship of the frequency shift and force strength has been given by Sader and Jarvis.[39]

The catalyst model was prepared by exposing a sputter-annealed TiO_2_ wafer to a Pt vapor source for 20 min. In the topography of Figure 2 a, four stacked terraces were separated by winding steps. The terrace width was 100 nm or more, and the step height was 0.3 nm. These features reproduced the topographic features of sputter-annealed TiO_2_(110) wafers observed in vacuum.[40-42] Nanometer-sized particles additionally appeared on the terraces. The height of the nanoparticles was smaller than the step height. The raw topography was duplicated and adjusted in contrast to identify nanoparticles, as shown in Figure 2 b–e. Twenty nanoparticles are identified and marked with circles in the four contrast-adjusted images. The topographic height of the marked particles was determined by analyzing cross sections. The height distribution was limited at 0.4 nm with an average height of 0.23 nm as shown in Figure 2 f.

**Figure 3:**
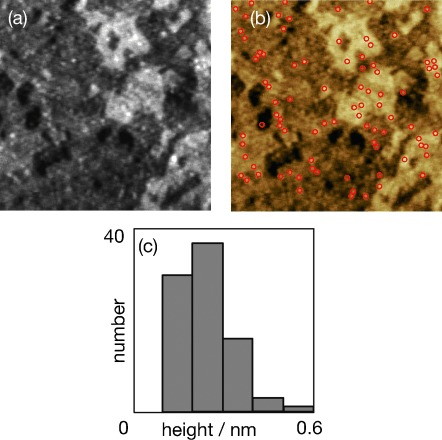
The topography of a catalyst model comprising TiO_2_(110) with deposited Pt observed in a N_2_ atmosphere. A sputter-annealed TiO_2_ wafer was exposed to the Pt source for 60 min. The raw topography (a) is in a gray scale. Platinum nanoparticles identified in the topography are marked with circles in (b). The height distribution of the identified particles is shown in (c). Image size: 300 nm square. Frequency shift set point: −220 Hz. Peak-to-peak amplitude: 4.8 nm. Quality factor of the cantilever oscillation: 400.

We assigned the nanoparticles to deposited Pt, because the number density increased on another catalyst model that was exposed to the Pt source for 60 min. Figure 3 a shows the raw topography of the catalyst model obtained after 60 min deposition. Eighty-seven nanoparticles were identified over two stacked terraces and marked in Figure 3 b. The topographic height distribution of the identified nanoparticles is presented in Figure 3 c. Comparing to the distribution shown in Figure 2 f, the average height shifted slightly to 0.25 nm with the top of the distribution at 0.6 nm. The average heights of 0.23–0.25 nm suggest a one- or two-atom-layer thickness of the nanoparticles on the two catalyst models. The layer-to-layer distance of Pt(111) is 0.23 nm based on the face-centered cubic lattice constant of 0.39 nm.

When Pt was deposited on TiO_2_(110) and imaged in a vacuum, not exposed to air, the deposited nanoparticles showed a height distribution ranging from 0.1 to 0.5 nm.[9] This finding shows that the particle height is insensitive to exposure to air and then N_2_. There was no sign of flattening or sharpening caused by the exposures. The lateral size of the nanoparticles was difficult to compare on different catalyst models. The lateral size of a nanometer-sized object is often presented in the convolution of the object and tip apex.

**Figure 4:**
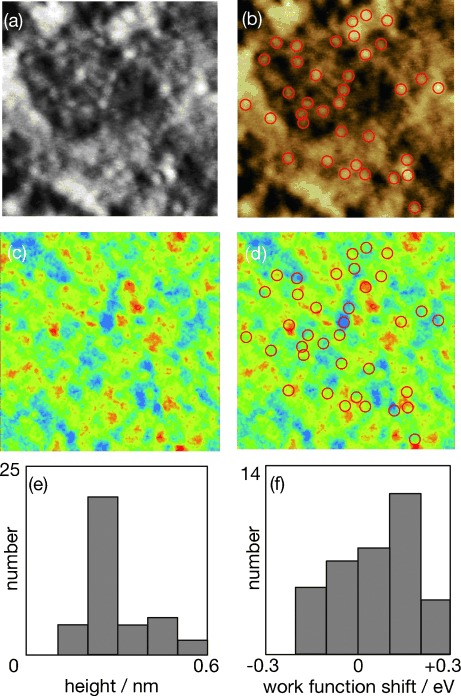
The topography (a) and work function distribution (c) of a catalyst model comprising TiO_2_(110) with deposited Pt. A sputter-annealed TiO_2_ wafer was exposed to the Pt source for 180 min and scanned in a N_2_ atmosphere. Thirty-six nanoparticles are identified in the topography (a). The identified nanoparticles are marked with circles in duplicated topography (b). The work function distribution is shown in (c), and the same distribution with marked nanoparticles is also shown (d). The topographic height and work function shift induced by the nanoparticles are summarized in (e) and (f), respectively. Frequency shift: −80 Hz. Peak-to-peak amplitude: 5.2 nm. Quality factor of the cantilever oscillation: 440. Image size: 100 nm square.

The local work function of Pt nanoparticles was observed on another catalyst model prepared by 180 min deposition. The number of nanoparticles increased accordingly. Figure 4 presents the topography and lateral distribution of work function simultaneously observed in the N_2_ environment of one atmospheric pressure. Thirty-six Pt nanoparticles were identified in the raw topography of Figure 4 a and marked in Figure 4 b. The topographic height distribution was not affected much by the increased deposition time, as shown in Figure 4 e. The average height was 0.30 nm.

Figure 4 c presents the work function distribution. Portions colored red showed larger work function relative to that of the TiO_2_ surface, whereas blue portions were of smaller work function. The nanoparticles identified in the topography are marked in the duplicated distribution of Figure 4 d. On the individual nanoparticles marked, the work function shift was determined relative to that of the TiO_2_ surface. Figure 4 f shows the summary of the particle-induced shifts of work function. Twenty-four nanoparticles presented positive shifts, and the other twelve showed negative shifts in a range of ±0.3 eV.

The work function shift distribution was broad across the origin. The median of the distribution was +0.15 eV. The broad distribution suggests heterogeneous properties of the Pt nanoparticles, although they are uniformly deposited in the vacuum, transferred in air, and exposed to the N_2_ environment. In contrast, Pt nanoparticles deposited and observed in the vacuum presented negative shifts proportional to the lateral size of the nanoparticles in the range from −0.1 to −0.5 eV.[9] The size-dependent, systematic shifts indicated the homogeneous nature of the nanoparticles when they were not in contact with air and N_2_. Oxygen, water, and CO_2_ may be adsorbed on the nanoparticles by exposing the catalyst model to air. Adsorbed species create additional dipole moments at Pt surfaces. Adsorption of different species leads to the different signs of work function shift. The heterogeneous shifts of work function may hence be ascribed to different manners of adsorption on the originally homogeneous Pt nanoparticles. This is a simple hypothesis explaining the observed results as a start for future considerations. Additional analysis techniques are required to complete the full picture of heterogeneous charge transfer across the Pt–TiO_2_ interface.

The major contribution of this study is demonstrating the ability of Kelvin probe force microscopy in observing catalyst-related nanostructures even in the presence of vapor environments. The microscope was placed in a N_2_ environment in the current study. The environment can be replaced with desired reactant vapor for catalyst applications, though care should be taken against possible corrosion of microscope materials.

## Conclusions

Platinum nanoparticles were vacuum-deposited on TiO_2_(110) to prepare a catalyst model. The lateral distribution of work function was observed over the Pt-deposited TiO_2_ surface in the presence of the N_2_ environment of one atmospheric pressure using an advanced Kelvin probe force microscope. Some nanoparticles presented positive shifts of work function relative to that of the TiO_2_ surface, while the others showed negative shifts. This result suggests that exposure to air and then N_2_ made the nanoparticles heterogeneous.

## Experimental Section

A commercial AFM instrument (SPM-9600, Shimadzu) was modified according to Fukuma et al.[37] with a low-noise cantilever deflection sensor and radio-frequency modulation of laser diode power. The total noise of the optical beam deflection was reduced to 7 fm Hz^−1/2^ in air. Imaging scans were done with doped silicon cantilevers (PPP-NCHR, Nanosensors). The nominal resonance frequency and spring constant of the cantilevers were 300 kHz and 42 N m^−1^, respectively. The bias voltage *V*_s_ of the sample was modulated relative to the tip by a peak-to-peak amplitude of 5 V at 3 kHz. The microscope was placed in a glove box.

Atomically flat (110) wafers of rutile TiO_2_ (10×10×0.5 mm^3^, Shinko-sha) were prepared in a UHV chamber equipped with an Ar ion gun (EX03, Thermo). A wafer was clamped with tungsten mesh on a sample holder, sputtered with Ar ions of 2 keV at room temperature (RT), and annealed in the vacuum at 900 K. The (1×1) order was checked by low-energy electron diffraction. The annealed wafer was cooled to RT and exposed to a heated Pt wire. The exposed wafer was removed from the chamber, transferred in air, and placed on the microscope in the glove box. The glove box was evacuated with a turbo molecular pump to 10^−1^ Pa and then filled with research-grade N_2_ gas of one atmospheric pressure. Imaging scans were conducted at RT in the N_2_ atmosphere.
